# Melanoma Disparities among US Hispanics: Use of the Social Ecological Model to Contextualize Reasons for Inequitable Outcomes and Frame a Research Agenda

**DOI:** 10.1155/2016/4635740

**Published:** 2016-08-29

**Authors:** Valerie M. Harvey, Charlene W. Oldfield, Jarvis T. Chen, Karl Eschbach

**Affiliations:** ^1^Department of Dermatology, Eastern Virginia Medical School, Norfolk, VA, USA; ^2^Hampton University Skin of Color Research Institute, Hampton, VA, USA; ^3^Department of Social and Behavioral Science, Harvard School of Public Health, Boston, MA, USA; ^4^University of Texas Medical Branch, Sealy Center on Aging, Galveston, TX, USA; ^5^University of Texas Medical Branch, Center to Eliminate Health Disparities, Galveston, TX, USA

## Abstract

Cutaneous melanoma is a significant public health concern, accounting for thousands of deaths annually in the US. Early detection and diagnosis are critical given the poor prognosis and limited treatment options of advanced-stage disease. While non-Hispanic whites have higher incidence rates of melanoma, Hispanics are typically diagnosed at later disease stages and suffer higher morbidity and mortality. Currently, there is a paucity of literature investigating the root causes underlying these trends among Hispanics. Given that Hispanics are the most rapidly expanding demographic segment in the US, it is essential for cancer control efforts to elucidate the major determinants of their poor melanoma outcomes. Herein, we use the social ecological model as a framework to explore the multitude of influences on melanoma disparities among Hispanics and provide recommendations for planning future studies and interventions.

## 1. Introduction

Cutaneous melanoma (CM) is a significant public health concern, accounting for approximately 10,000 deaths annually in the United States (US) [1]. The poor prognosis and limited treatment options of advanced-stage disease make early detection and diagnosis critical. Although the incidence of melanoma is greatest in non-Hispanic whites (NHWs), numerous studies have shown that Hispanics diagnosed with melanoma are more likely to experience worse outcomes [2–7]. Surveillance, Epidemiology, and End Results data reveal that Hispanics diagnosed with melanoma are 2.4 times (age-adjusted odds ratio (OR), confidence interval (CI) 1.89–3.05) more likely to present with stage III disease [6] and 3.64 times more likely (CI 2.65–5.0) to have distant metastases than non-Hispanic whites (NHWs) [3, 4, 8–11]. Moreover, while the proportion of those with local-staged melanoma has increased significantly among NHWs, an opposing trend has been reported among Hispanic men living in California who were found to have thicker primary tumors at diagnosis [12]. Factors in the late presentation of CM in Hispanics may include lack of awareness and knowledge [6], lower rates of self-examinations and physician performed skin examinations [7], and differences in tumor biology [6]. For cancer control efforts to succeed, we must better understand the major causes of advanced presentation of melanoma in Hispanics (Hispanics and Latinos) who represent the most rapidly expanding demographic segment in the US.

The Hispanic category is multiethnic, encompassing populations with distinct national origins. Census Bureau data shows 55 million Hispanics living in the US, comprising of 35 million Mexican Americans, 8.7 million Puerto Ricans (including 3.4 million in Puerto Rico), 2 million Cubans, and 1.8 million Dominicans [13]. The recent immigration of over 8 million Hispanics from Central and South America has increased the diversity of the Hispanic population and even displaced Cubans as the third largest group [13]. These national origin groups are increasingly diverse, in nativity, primary language, acculturation, education, and occupation. Understanding the contributors to late-stage melanoma presentation in this heterogeneous group provides an opportunity to explore the effects of immigration and acculturation on determinants of cancer risk and outcomes. The objective of this article is to provide a framework for studying melanoma disparities in Hispanics and to provide recommendations for a research agenda to facilitate future research and interventions.

## 2. Conceptual Framework

**Figure 1:**
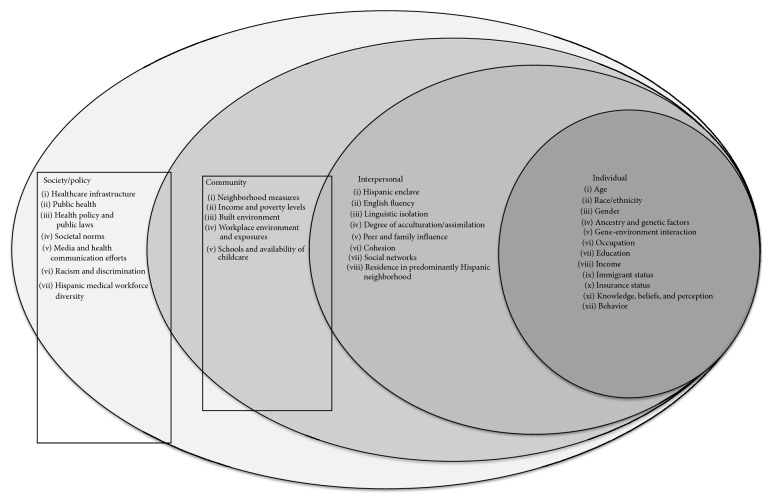
Social ecological model [88].

The social ecological model (SEM) provides a dynamic and integrative framework for analyzing social and spatial variations in human health and disease [14, 15] (Figure 1). At the model's core lies the concept that health outcomes result from a unique combination of personal, interpersonal, and community attributes, thereby contextualizing the reciprocal interdependence of individuals and their environment. The individual level identifies biological and personal factors, including age, gender, genetics, knowledge, and behavior. The interpersonal level looks at how relationships with peers, partners, families, and social networks influence health. The community level examines settings such as the neighborhoods, schools, and workplaces within which disease occurs and seeks to identify the characteristics of these settings that promote or prevent disease. The policy/societal level investigates the broader structural determinants such as health system infrastructure, health care access, health care resources and utilization, health policies, workforce diversity, and societal norms. While numerous studies have documented connections between individual health outcomes and the influence of these social and spatial determinants [16–22], few have used this paradigm to understand the reasons for disparities in melanoma outcomes [23].

## 3. Individual Determinants

Individual attributes, including age, race/ethnicity, gender, beliefs/attitudes, behavior, and health insurance status are thought to impact cancer outcomes [24, 25]. While age-adjusted incidence rates of CM among Hispanics (4.8/100,000 for men and 4.5/100,000 for women) are lower than those for NHWs (33.6/100,000 and 20.2/100,000 for men and women, resp.) [26], Hispanics are more likely to be diagnosed at a more advanced stage and have thicker tumors than their non-Hispanic white counterparts [4, 6, 27]. Moreover, Hispanics have an increased mortality [4, 5]. The clinical presentation of CM also differs across race and ethnicity, with Hispanics having a higher incidence of lower extremity involvement (25% versus 9%;* P* < 0.001) [5], and a greater frequency of the acral lentiginous histological subtype than NHWs [5, 6]. The reasons for phenotypic variability are not entirely clear but may include genetic factors, gene-environment interaction, and/or inherent differences in the kinetics of tumor growth.

### 3.1. Health Insurance

Notably, Hispanics have the highest uninsured rate of any other racial/ethnic group in the US [28]. This is due in part to low-wage jobs without employer-based health benefits. Health insurance status has been shown to be a determinant of disparities in melanoma outcomes [29–31]. Intermittent and newly enrolled Medicaid CM patients were 2–13 times more likely, respectively, to have late-stage melanoma than those not on Medicaid. Medicare and Medicaid patients with CM were less likely than their privately insured counterparts to undergo sentinel lymph biopsies [29], indicating that publicly insured individuals may be understaged and possibly inadequately treated. Insurance may also attenuate disparities. For example, Hispanics enrolled in Medicare health maintenance organizations (HMOs) were diagnosed with CM at earlier stages and had decreased mortality than their age-matched controls in a Medicare fee-for-service program [32]. This observation may be in part due to the “HMO effect,” meaning that those enrolled in an HMO will have more points of contact with their primary care provider and may therefore be more likely to undergo preventive services such as skin cancer screenings [32].

### 3.2. Knowledge and Awareness

Knowledge may be an important predictor of preventive behaviors and use of screening services for melanoma. Temoshok et al. found that low levels of CM knowledge and understanding were associated with greater tumor thickness at diagnosis [33]. Studies show that Hispanics have less melanoma knowledge and awareness than NHWs [34–38]. In general, Hispanics also have a low perception of personal cancer risk [34, 35, 39, 40]. Hispanics also believe they can do little to reduce their risk of skin cancer, primarily because the many recommendations make it hard for them to know which ones to follow [41]. Moreover, Hispanics are thought to ascribe to fatalism about skin cancer prevention [41, 42]. This passivity may explain why they are also less likely to seek medical care if they develop a suspicious skin lesion. Current evidence, however, is insufficient to determine how much these knowledge gaps contribute to CM inequities [43].

### 3.3. Behavior

Individuals who are knowledgeable and aware of the perils of melanoma are more likely to perform skin self-examinations [38, 44]. In general, compared to other racial and ethnic groups, Hispanics are less likely to comply with cancer screening guidelines [45] and have significantly lower rates of skin self-examination [27, 35, 46–49] and physician-assisted skin examination [49–51] than NHWs. Among Hispanics, factors associated with skin self-examinations and receipt of physician-assisted skin examinations include greater US acculturation, older age, an increased number of melanoma risk factors, physician recommendations [7, 40, 52], fewer skin self-examination barriers, [52] country of origin [7], tanning indoors, greater knowledge about skin cancer, greater perceived skin cancer severity, a low worry of skin cancer, and added physician-assisted skin examination benefits [52]. Hispanics cite lack of awareness of self-examination and lack of knowledge of how to conduct such an examination as primary reasons for not performing skin self-examinations [35, 40, 48]. The primary reasons for not receiving physician-assisted skin examinations were inadequate time with the physician and not knowing to ask or how to ask for an examination [40].

### 3.4. Occupation

A significant proportion of Hispanics work in outdoor occupations such as agriculture/landscaping, construction, maintenance, and transportation, all of which are associated with significant UV radiation and chemical exposure [53]. There is a paucity of data exploring occupational influences on skin cancer risk in this population. Day et al. found that a majority of outdoor Hispanic workers reported never wearing sunscreen, and 44% of study participants reported ever having a sunburn [54]. Similarly, 91% of migrant farmworkers in North Carolina reported never wearing sunscreen and had low levels of knowledge about melanoma [54, 55]. Melanoma risk has also been inconsistently linked to exposure to various chemicals, including arsenic and various pesticides [56]. Dennis et al. found significant associations between melanoma and several classes of pesticides in a large cohort from the Agricultural Health Survey [56]. Future longitudinal studies quantifying UV exposure as well as exposure to other environmental toxins in high risk occupations for skin cancer and melanoma development are warranted, although the low incidence of CM in this group may limit the feasibility of this type of prospective investigation [55].

### 3.5. Immigration Status

More than 19 million or 35% of the more than 55 million Hispanics in the US (excluding Puerto Rico) are foreign-born [57]. Of these, nearly 13 million or 23% are not US citizens [57]. The different Hispanic subgroups have very different settlement patterns and migration histories in the US. Virtually all Puerto Ricans, including the island-born, are native-born US citizens. More than two-thirds of Mexican Americans in the US were US-born. By contrast, all other major national origin groups are majority foreign-born. Each of the individual risks discussed above—insurance status, health related knowledge and behaviors, and occupational exposures—varies partially in relation to immigrant status. For example, in 2014, 14% of US-born Hispanics reported no current health insurance coverage, compared to 8% of all NHWs and 14.5% of all non-Hispanic Blacks. Among Hispanic immigrants who were citizens, 17% did not have health insurance coverage. However, more than half of noncitizen immigrants did not have health insurance, accounting for a majority of the uninsured Hispanics.

This heterogeneity provides opportunities for understanding and exploring how the genome, environment, and cultural factors contribute to CM development. It also creates important methodological challenges. The presence of a large undocumented population creates concerns about completeness of census counts supplying the denominators of incidence and mortality rates. Incidence counts may also be suppressed if a large undocumented and uninsured population has impaired access to screening and diagnosis. Mortality may be under ascertained in an immigrant population because of health selective return migration out of the US after a CM diagnosis. These issues have been recognized in the epidemiological literature, but empirical investigation of their net impact on the data has been limited [45].

## 4. Interpersonal Determinants

The SEM also considers how interpersonal determinants, or relationships with peers, family members, and the immediate social milieu influence health outcomes. Acculturation, the process by which immigrants and their families adopt the language, attitudes, behaviors, and norms of their host country, has been associated with behavioral changes in relation to skin cancer risk among Hispanics [55, 58–60]. US cultural norms favor sunscreen use and sun tanning more than Hispanic cultural norms [58]. Acculturation among Hispanics has been linked to higher perceived benefits of exposure to UV radiation [60], less worry about skin damage [60], higher rates of sunbathing [59], higher rates of indoor tanning [59], and an increased risk of sunburns [55]. One study found that more than half of acculturated Hispanics reported having sunburns in the past year, as compared to just 1 in 3 of less acculturated Hispanics [55]. Importantly, sun-protective behaviors vary by country of origin. Individuals from Central/South America, Puerto Rico, and Cuba reported lower rates of having a sunburn and decreased usage of sun-protective clothing than those from other Hispanic/Latino countries [55]. These examples highlight the importance of considering the diverseness of the Hispanic population when investigating health outcomes [55].

## 5. Community Determinants

Neighborhood measures of income, poverty, economic opportunity, and level of educational attainment are community factors shown to influence health and disease [5, 9, 10, 61, 62]. Little research exists on the community determinants of melanoma outcomes in Hispanics; what exists relies on composite measures from population-based cancer registry data. This approach makes it difficult to tease apart Hispanic subgroups and to decipher which determinants, either singly or in combination, result in melanoma inequities. Among NHWs, indicators of high socioeconomic status (SES) are associated with higher melanoma incidence rates; however, among Hispanics, 66% of the melanoma burden occurs within middle and low SES groups [5]. This opposing SES gradient in incidence suggests the existence of a unique set of exposure risks particular to Hispanics. Irrespective of race, ethnicity, or sex, patients from lower SES strata were more likely to present with thicker tumors (>2 mm) at diagnosis; however, the association of thicker tumors and low SES was most profound among Hispanic men (relative risk [RR] 2.18; confidence interval [CI] 1.73–2.74) and women (RR 1.98; 1.55–2.51), suggesting Hispanics may be disproportionately burdened by barriers to screening and care due to poverty [5]. In Florida, residence in an impoverished neighborhood was found to independently predict late-stage clustering of CM in Hispanics [63].

### 5.1. Education

Education helps individuals recognize the signs and symptoms that indicate the need for prompt medical attention and help them navigate through the health care system [64]. Cokkinides et al. examined age-adjusted mortality rate trends by level of educational attainment; they found that while melanoma mortality rates fell for highly educated individuals (greater than or equal to 13 years of education) over the 10-year study period, mortality for less-educated melanoma patients increased (*P* = 0.17) [65].

### 5.2. Access to Preventive Measures

Sunscreen availability differs across neighborhoods. To date, two studies have compared sunscreen accessibility within Hispanic and non-Hispanic communities. In Chicago, Hernandez et al. found that twice as many stores sold sunscreen in non-Hispanic neighborhoods with greater product diversity compared to Hispanic neighborhoods [66]. However, Sprague et al. were unable to replicate these results in Los Angeles, finding no significant difference in availability or diversity of sunscreen [67]. Further research is needed to elucidate the potential, if any, differences in sunscreen availability as well as the barriers to its usage and attainment that may influence CM outcomes.

## 6. Society and Policy Determinants

At the macro level, inequitable cancer health outcomes occur in the context of upstream societal and political factors. These elements include public/health policy and laws, health service infrastructure (financing, access, and quality), and societal norms (discrimination, racism, etc.).

### 6.1. Public Insurance Infrastructure

Hispanics face multiple barriers that impede access to quality healthcare. Although the rate of uninsured Hispanics among working age adults declined from 40% to 34% in 2014 with the implementation of the Patient Protection and Affordable Care Act (PPACA), Hispanics still have the highest uninsured rates of all major US racial or ethnic groups [68]. While half of uninsured Hispanics qualify for expanded Medicaid under PPACA, only 29 states and the District of Columbia enforce this provision [68]. Notably, Florida and Texas, two states which together account for a large proportion of the Hispanic populace, are among the states not participating in the Medicaid expansion. Among nonparticipating states, Hispanics continue to hold uninsured rates approaching 46% [68].

### 6.2. Immigration Status and Insurance

As discussed above, the low rate of health insurance coverage pertains primarily to noncitizen immigrants, of whom just less than half report current health insurance coverage. Barriers are particularly high for the estimated 8 million undocumented immigrants from Latin America [69]. By law, undocumented immigrants are not qualified to attain health insurance through either the federal Marketplace [70] or Medicaid [71]. Medicaid also enforces a 5-year waiting period which may be waived for children, pregnant women, and certain “qualified noncitizens” (green card holders, lawful permanent resident, etc.) [70]. Overall, these policies impede primary care access. Over one-quarter of Hispanic adults lack a consistent primary care provider and are twice as likely as non-Hispanic blacks and three times as likely as NHWs to not have a regular healthcare provider [72]. Inconsistent primary care and dermatologic access have been linked to advanced melanoma at presentation [73] and decreased melanoma survival [74].

### 6.3. Hispanic Health Professional Shortage

Numerous studies have identified provider behaviors and bias as key contributing factors of health inequities, suggesting that cultural differences between patients and their providers impact healthcare outcomes [75–77]. Among Hispanics, race concordance is an important predictor of greater satisfaction with overall healthcare quality [78]. Hispanic physicians are more apt to provide medical care to minorities, low income, and non-English speaking patients and play a critical role in improving access to care among the underserved. Unfortunately, Hispanics are grossly underrepresented among the US physician workforce, comprising only 4.6% of US medical school graduates in 2014 [79].

### 6.4. Health Communication and Educational Campaigns

Health communication campaigns help shape health perceptions and risk behaviors, including those associated with melanoma. Studies demonstrate that mass media campaigns about health influence risky behaviors [80]. Previously, mass media campaigns have successfully increased screenings for cervical [81–83] and breast cancer [84]. Despite the plethora of public messaging on melanoma, most is geared towards middle aged whites [12, 85] with little being tailored towards the Hispanic population [12, 59, 85, 86]. While half of US adults report cancer-related internet searches on cancer [87], Hispanics may use it less than whites. Hay et al. found that nonwhite adults are more receptive to health information obtained via televised news [37]. Similarly, the Pew Hispanic Center and the Robert Wood Johnson Foundation found that 68% of Hispanic adults report obtaining their health information via television [72].

## 7. Conclusions and Recommendations

Hispanics diagnosed with melanoma typically present with advanced stages of disease and experience higher melanoma-specific mortality rates than NHWs. Yet, huge knowledge gaps exist regarding the contributors and solutions to melanoma disparities among this fast growing, understudied segment of the US population. In Table 1 we present research questions and strategies for promoting melanoma outcome equity, based on SEM level of influence. This approach highlights the need for a multidisciplinary approach spanning multiple levels of influence. Although the absolute frequency of melanoma is relatively low within the Hispanic population, melanoma studies in Hispanics (given their diverse genetic ancestry and migration) provide a unique opportunity for research to explicate melanoma etiology, benefiting Hispanics and non-Hispanics alike.

## Figures and Tables

**Table 1:** Research questions and strategies by social ecological model levels of influence [89].

Research questions and strategies by SEM levels of influence		
Level of influence	Sublevel of influence	Future research questions and strategies
Society/policy	(i) Healthcare infrastructure (ii) Public health (iii) Health policy and public laws (iv) Societal norms (v) Media and health communication efforts (vi) Racism and discrimination (vii) Hispanic medical workforce diversity	(i) How does health system infrastructure impede access to dermatologic care among Hispanics? (ii) How can different media platforms be employed to increase knowledge and awareness among Hispanic subpopulations and influence CM societal norms? (iii) What are the most effective methods of dissemination of CM education? (iv) Can CM educational interventions designed for Hispanics influence outcomes? (v) How does racism/discrimination influence CM outcomes? (i) Training of physician extenders (and screen patients for skin cancer and melanoma. (ii) Implement policies that remove barriers to access of primary and specialty care. (iii) Diversification of the medical and dermatologic workforce to increase the representation of Hispanic care providers. (iv) Development of culturally and linguistically appropriate melanoma educational campaigns targeted to Hispanic subgroups. (v) Collaboration between Hispanic community stakeholders and professional medical organizations to facilitate dissemination of information/research findings to lay community.

Community	Neighborhood measures (i) Income/level of poverty (ii) Built environment (iii) Workplace environment and exposures (iv) Schools and availability of childcare	Are there unique environmental risk factors (aside from UV exposure) that increase CM risk among Hispanics? Develop longitudinal cohort studies quantifying workplace exposures and their impact on CM outcomes in Hispanics.

Interpersonal	(i) English language fluency (ii) Residence in predominantly Hispanic neighborhood (iii) Degree of assimilation (iv) Cohesion (v) Social networks (vi) Peer and family influence	What cultural factors contribute to CM risk/mortality? Do these factors vary by Hispanic country of origin? Are there components of Hispanic culture that influence participation in skin cancer screening or seeking medical care for skin conditions? How does melanoma risk vary by degree of acculturation? Develop more precise constructs to measure effects of acculturation. Development of community-based participatory research studies in which Hispanic stakeholders are included in early stages of study design.

Individual	(i) Age (ii) Race & ethnicity (iii) Gender (iv) Ancestry and genetic factors (v) Gene-environment interaction (vi) Immigration status (vii) Knowledge and behavior (viii) Insurance status (ix) Occupation (x) Health insurance status (xi) Income (xii) Education	What is the phenotypic profile of Hispanic patents with CM? Are kinetics of melanoma progression and metastasis different among Hispanics? How do CM risk and mortality vary by Hispanic subgroup? How does CM risk vary with immigration status and duration of time in US? How do fatalism and its impact on skin cancer perceptions vary between Hispanic subgroups? What role, if any, does fatalism play in CM inequities? How can melanoma knowledge and awareness be increased amongst Hispanic subgroups? Develop genetic studies (AIMS/GWAS) across Hispanic subgroups to understand the genetic basis for melanoma risk. Identification of biomarkers for melanoma disease progression within Hispanics. Case control studies of Hispanics with and without melanoma to help identify unique risk factors amongst this group. Efforts to increase recruitment of Hispanics melanoma patients into clinical trials. Transdisciplinary studies to understand the interlink between biological and social factors.
